# Nipah Virus Infection Outbreak with Nosocomial and Corpse-to-Human Transmission, Bangladesh 

**DOI:** 10.3201/eid1902.120971

**Published:** 2013-02

**Authors:** Hossain M.S. Sazzad, M. Jahangir Hossain, Emily S. Gurley, Kazi M.H. Ameen, Shahana Parveen, M. Saiful Islam, Labib I. Faruque, Goutam Podder, Sultana S. Banu, Michael K. Lo, Pierre E. Rollin, Paul A. Rota, Peter Daszak, Mahmudur Rahman, Stephen P. Luby

**Affiliations:** Author affiliations: icddr,b, Dhaka, Bangladesh (H.M.S. Sazzad, M.J. Hossain, E.S. Gurley, S. Parveen, M.S. Islam, L.I. Faruque, G. Podder, S.P. Luby);; Institute of Epidemiology, Disease Control and Research, Dhaka (K.M.H. Ameen, S.S. Banu, M. Rahman);; Centers for Disease Control and Prevention, Atlanta, Georgia, USA (M.K. Lo, P.E. Rollin, P.A. Rota, S.P. Luby);; EcoHealth Alliance, New York, New York, USA (P. Daszak)

**Keywords:** Nipah, encephalitis, outbreak, Nipah virus, NiV, nosocomial, healthcare-associated infection, corpse, Bangladesh, viruses, burial practices, PPE, personal protective equipment, health care workers, transmission

## Abstract

Active Nipah virus encephalitis surveillance identified an encephalitis cluster and sporadic cases in Faridpur, Bangladesh, in January 2010. We identified 16 case-patients; 14 of these patients died. For 1 case-patient, the only known exposure was hugging a deceased patient with a probable case, while another case-patient’s exposure involved preparing the same corpse for burial by removing oral secretions and anogenital excreta with a cloth and bare hands. Among 7 persons with confirmed sporadic cases, 6 died, including a physician who had physically examined encephalitis patients without gloves or a mask. Nipah virus–infected patients were more likely than community-based controls to report drinking raw date palm sap and to have had physical contact with an encephalitis patient (29% vs. 4%, matched odds ratio undefined). Efforts to prevent transmission should focus on reducing caregivers’ exposure to infected patients’ bodily secretions during care and traditional burial practices.

## Abstract 2

Particularly vulnerable are health care workers who do not use personal protective equipment and persons who do not wash hands after traditional burial practices.

In Bangladesh, 135 probable or confirmed cases of Nipah virus (NiV) infection in humans were identified from 2001 through 2008; 98 (73%) were fatal (1). Drinking raw date palm sap, contaminated by NiV from urine or saliva of *Pteropus* spp. fruit bats, has been identified as a vehicle for transmission of NiV to humans in Bangladesh (2,3). NiV, an RNA paramyxovirus (4), was isolated from human respiratory secretions, saliva, and urine during the outbreaks (5,6). Outbreak investigations in Bangladesh and India have repeatedly implicated person-to-person transmission of NiV, including health care–associated transmission in the Siliguri, India, outbreak in 2004 (7–10). However, to our knowledge, no evidence of NiV transmission to health care workers had been confirmed in Bangladesh (11).

In the area where NiV outbreaks have been repeatedly identified (Figure 1), the Institute for Epidemiology, Disease Control and Research (IEDCR) of the Government of Bangladesh, in collaboration with icddr,b (formerly the International Centre for Diarrhoeal Disease Research in Bangladesh) is conducting hospital-based encephalitis surveillance. To detect outbreaks of NiV infection, the surveillance system identifies sporadic NiV cases during January–March and clusters of encephalitis patients throughout the year. On January 14, 2010, two cousins living in the Faridpur District in Bangladesh (Figure 1) were admitted to Faridpur Medical College Hospital (FMCH) with fever and altered mental status. A team from IEDCR and icddr,b initiated an investigation on January 15, 2010. The objectives of the investigation were to identify the cause of the outbreak and to detect sporadic cases of NiV infection.

**Figure 1:**
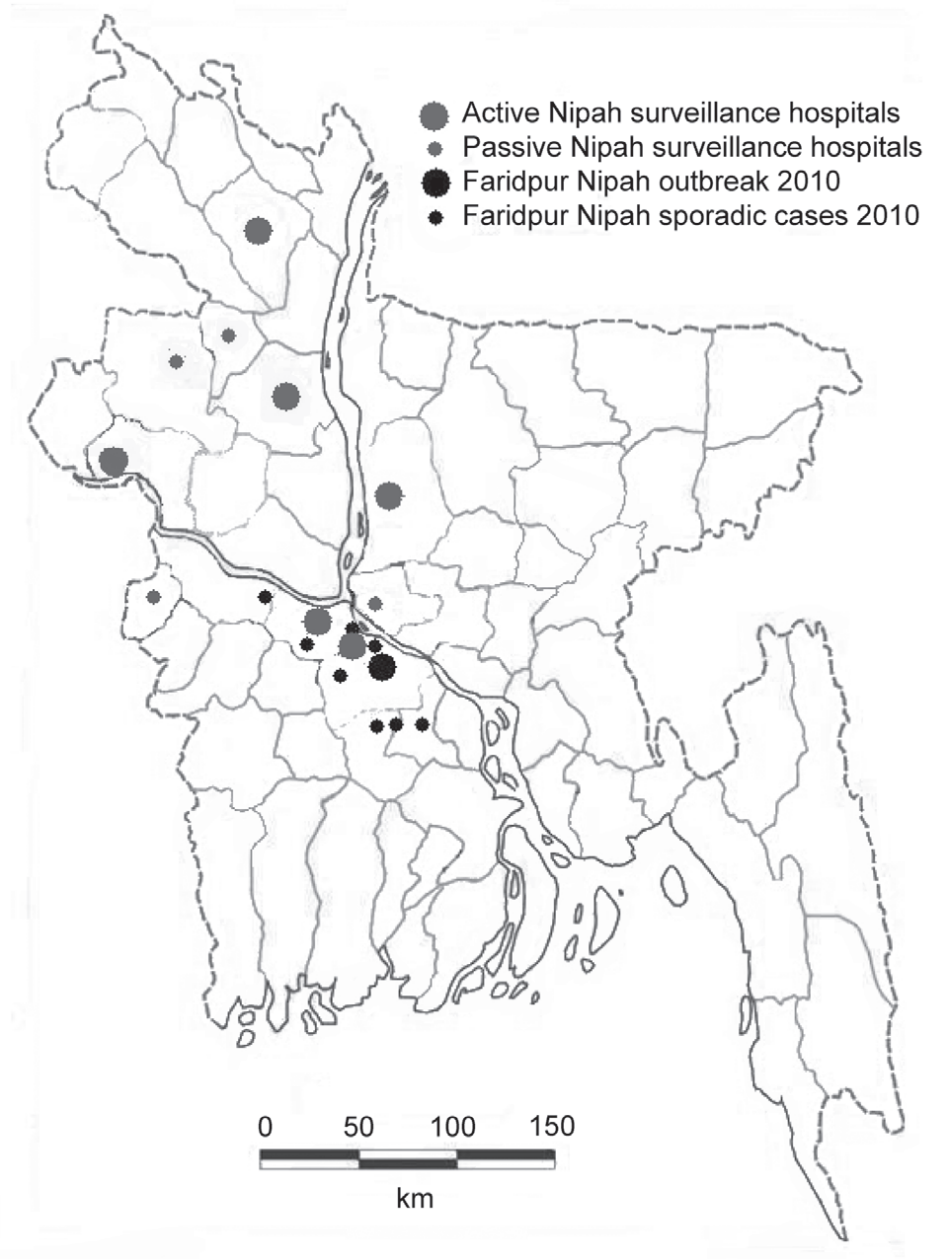
Surveillance hospitals and locations of outbreak clusters and sporadic cases of Nipah virus infection, Bangladesh, 2010.

## Methods

### Case Identification and Specimen Collection

#### Hospital Surveillance

Surveillance physicians maintained a registry of patients who sought treatment with fever or with history of fever with axillary temperature >38.5°C (101.3°F), altered mental status, new onset of seizures, or a new neurologic deficit, either diffuse or localized to the brain (12). The physicians collected whole blood, cerebrospinal fluid, a throat swab specimen, and urine samples from patients admitted during January–March 2010 in 3 of the 6 active surveillance hospitals (Faridpur, Rajshahi, and Rangpur Medical College Hospitals) and during January–April 2010 at Faridpur Medical College Hospital. These samples were stored in liquid nitrogen in local hospitals and transported biweekly to the IEDCR laboratory.

#### Outbreak Area

We defined possible case-patients as persons with acute onset of fever living in the outbreak area with onset of illness from January 7 through January 28, 2010. We identified possible case-patients by conducting door-to-door visits of all homes and contacting local physicians in the affected villages. We defined probable case-patients as persons who met the possible case definition and who had new onset of altered mental status or new onset of breathing difficulty. We defined case-patients with laboratory-confirmed NiV infection as those with detectable serum IgM against NiV. To assess asymptomatic NiV infection in the outbreak community, we asked community members who had close physical contact or had shared date palm sap from the same pot with probable or confirmed case-patients within the preceding month, to provide a blood specimen for serologic testing.

We obtained a clinical history and information about exposures during the month before illness from each probable and confirmed case-patient. Friends, relatives, and neighbors of deceased or unconscious case-patients served as proxy informants for interviews.

The investigation team collected acute-phase and subsequent whole blood specimens from possible case-patients. Samples were centrifuged in the local government community clinic, and the separated serum was stored and transported to the IEDCR laboratory in liquid nitrogen. Samples were stored at −70°C.

### Laboratory Testing

We tested serum samples at IEDCR with an IgM capture enzyme immunoassay that detects NiV IgM (13). We shipped an aliquot of serum, cerebrospinal fluid, throat swab specimens, and urine from patients with probable and confirmed cases of NiV infection and from those with IgM against NiV to the Centers for Disease Control and Prevention (CDC), Atlanta, Georgia, USA, for confirmatory testing.

### In-Depth Interviews

We conducted in-depth interviews with families of case-patients to explore history of illness and exposures of case-patients. We also had informal discussions with neighbors of case-patients to explore possible modes of transmission.

### Case–Control Study

We conducted a matched case–control study to identify risk factors for transmission of NiV. Persons with probable and confirmed cases from the outbreak and with sporadic cases identified from surveillance were considered to be case-patients. The field team selected 4 neighborhood controls for each case-patient, starting from the fourth closest courtyard to the case-patient’s residence. The courtyards where other case-patients resided were excluded. In each courtyard, only the household closest to the main entry was selected. The age of every person in the selected household was recorded. Then a control (only 1 from each courtyard), whose age was closest to the case-patient’s age, was selected. If that control was absent during the first visit, the team tried 3 times to reach the control. If the team was unsuccessful, no control was selected from that household. This process was repeated at the next closest courtyard household until we had selected the required number of controls.

All case-patients, except 1, were either too sick or too confused to respond or had died, so the field team selected multiple appropriate proxy respondents for interview. We used standardized, structured questionnaires in the Bengali language.

### Statistical Analyses

To estimate the association between each exposure and NiV infection, we calculated the matched odds ratio (mOR). We used conditional logistic regression and considered any association to be statistically significant if the p value was <0.05. We analyzed data in STATA 10 (Stata Corp., College Station, TX, USA).

### Ethics Approval

Legal guardians of study participants and healthy adult participants provided informed verbal consent for participation in this investigation. The Ethical Review Committee of icddr,b reviewed and approved the protocol for NiV surveillance and outbreak investigation.

## Results

### Cases

#### Hospital Surveillance

During January–April 2010, surveillance physicians from the 6 hospitals reported 328 meningoencephalitis case-patients. Of these, 106 (32%) were reported by surveillance physicians in Faridpur, including 4 outbreak case-patients. Ninety-seven (92%) serum samples were collected at the Faridpur surveillance site. Of these, 12 had IgM against NiV (12%), including 4 from the outbreak area.

#### Outbreak

**Figure 2:**
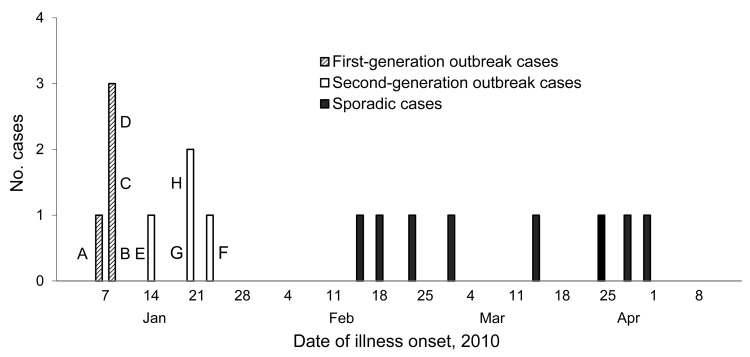
Nipah virus infection cases, Faridpur, Bangladesh, 2010. A–H indicate specific case-patients.

The outbreak investigation team identified 100 possible NiV-infected persons with febrile illness in the outbreak area during January 5–28, 2010. Of these, 68 persons gave a blood sample for NiV laboratory diagnosis. One person’s sample was positive for IgM against NiV and that person was classified as a confirmed NiV-infected case-patient. We collected 4 blood samples positive for IgM against NiV from 7 probable case-patients. The remaining 3 case-patients died before blood samples were collected. We defined these persons as probable NiV-infected case-patients. All but 1 case-patient had altered mental status, and two thirds of case-patients had breathing difficulty (Table 1). Seven (88%) of 8 persons who met the case definition for probable or confirmed NiV infection died. The onset of illness for all of case-patients occurred within 16 days of exposure. Four cases constituted an initial peak, and a second-generation outbreak of 4 cases appeared after contact with the initial case-patients (Figure 2).

**Table 1:** Demographic and clinical features of outbreak and sporadic case-patients with encephalitis caused by Nipah virus infection, Faridpur, Bangladesh, 2010*

Feature	First-generation outbreak, n = 4	Second-generation outbreak, n = 4	Sporadic, n = 8	All, n = 16
Median age, y (range)	28 (10–45)	55 (32–60)	23 (4–45)	35 (4–60)
Male sex	1 (25)	3 (75)	5 (63)	9 (56)
Clinical features				
Fever	4 (100)	4 (100)	8 (100)	16 (100)
Altered mental status	4 (100)	3 (75)	8 (100)	15 (94)
Unconscious	4 (100)	2 (50)	8 (100)	14 (88)
Difficulty breathing	2 (50)	3 (75)	7 (88)	12 (75)
Headache	4 (100)	2 (50)	4 (50)	10 (63)
Vomiting	4 (100)	1 (25)	3 (38)	8 (50)
Convulsion	3 (75)	1 (25)	3 (38)	7 (44)
Case-fatality rate	4 (100)	3 (75)	7 (88)	14 (88)
Median days (range) from onset of illness to death	7 (4–8)	6 (3–7)	4 (4–17)	5 (3–17)†

*Values are no. (%) case-patients except as indicated. †n = 14, all of whom died.

### Qualitative Findings on Exposure Histories

#### Sporadic Case-patients

Among 7 patients with sporadic cases of NiV infection, 6 of whom died, 5 had a history of drinking raw date palm sap in the month preceding illness onset, and 2 of these 5 were harvesters of date palm sap. One case-patient attended to a family member who was hospitalized at FMCH with an illness unrelated to encephalitis for 3 days, 4 days before the onset of illness. While staying in the hospital, he spent the days talking with and caring for other patients and slept alongside them.

Another case-patient was a physician in training at FMCH. From January 1 through February 28, 2010, he worked in the adult medicine and pediatric wards and clinically managed cases of meningoencephalitis in 14 patients, including 3 with confirmed NiV infection and other severely ill febrile patients who had not been enrolled in NiV surveillance. He performed physical examination, intravenous canalization, and nasogastric intubation for 1 confirmed case-patient in the adult medicine unit >1 month before the onset of his illness. He also similarly treated the condition of 2 confirmed NiV case-patients in the pediatric unit 4 days before his onset of illness. A handwashing station for physicians did not exist in either the adult medicine or pediatric wards, and his colleagues reported that he did not use gloves or a mask during patient care or wash his hands after patient care. His illness began on February 28, 2010, with fever and myalgia. Subsequently, he experienced coughing, respiratory distress, convulsions, altered mental status, and loss of consciousness. He died on March 6, 2010. The duration between onset of illness of the physician and his contact with 2 known NiV-infected case-patients in the adult medicine ward was 32 days, and the time from his contact with the 2 children with NiV infection to illness was 2–4 days. Two NiV genomic sequences, obtained from 2 of 14 meningoencephalitis patients that the physician had been in contact with, did not match the NiV genomic sequence of the physician’s isolates (14).

#### Outbreak Case-patients

Case-patient A, a 45-year-old man with a probable case of NiV infection, lived under a *Pteropus* bat roost. He drank raw date palm sap from his own date palm tree during the last week of December 2009, 1 week before the onset of illness on January 7, 2010. He had fever, headache, and myalgia, which progressed over 6 days to drowsiness, convulsions, confusion, unconsciousness, and death.

On January 7, three neighbors of case-patient A, from a single family (case-patients B, C, and D), bought and drank raw date palm sap from the village date palm sap harvester. All became ill on January 9, within hours of each other. None had any history of contact with any person who had symptoms similar to encephalitis. Probable case-patients A and B died on January 13; confirmed case-patients C and D died on January 16 and 17 (Figure 2).

Among those with second-generation cases, case-patient E (confirmed case) was the wife of case-patient A and was involved in feeding, comforting, and transporting her husband to FMCH and in bringing his dead body back home on January 13. She cleaned saliva her husband using her bare hands and did not wash her hands afterwards. She also drank raw date palm sap with case-patient A in last week of December and did not get ill. On January 15, 2010, a fever developed, but she did not have altered mental status. She recovered on the fourth day of illness and was a confirmed case-patient who survived. Case-patient F (confirmed case) was a friend of case-patient A, who frequently visited case-patient A during case-patient A’s illness. When case-patient A’s condition deteriorated, case-patients E and F took him to the hospital. Case-patient A had to be supported by case-patient F, and the distance between the faces of case-patients A and F was <1 foot while case-patient A was coughing, salivating, and having difficulty breathing. While bringing the dead body back from hospital by bus, case-patient A’s head rested on the thigh of case-patient F. Case-patient G (confirmed case) was the uncle of case-patient A who lived in the same village, and he never visited case-patient A during his illness. He had no contact with any other encephalitis case-patient. He arrived only after case-patient A died and had caressed the head of the corpse before the ritual corpse bathing. Case-patient H (probable case), was a neighbor of case-patient A, who did not come into contact with case-patient A during his illness. He carried out the Muslim practice of ritual purification by cleaning the body and washing it. He used pieces of cloth to clean the body orifices (anus, urethra, oral and nasal secretions) with an ungloved hand. He then washed the entire corpse with water. He took a bath 1 hour after the ritual cleansing. Case-patient G came in contact with the corpse of case-patient A 3 hours after case-patient A’s death, and case-patient H came in contact with the corpse of case-patient A 6 hours after case-patient A’s death. None of these 3 case-patients (F, G, and H), who cared for case-patient A before or after his death, had drunk raw date palm sap in the preceding month. Their illnesses began 8–12 days after contact with case-patient A, they had symptoms similar to those of case-patient A, and they died on January 27 or January 28, 2010, after 4–8 days of illness.

### Case–Control Study

The field team enrolled 15 case-patients and 58 controls (Table 2). The mean age of the case-patients and controls was similar (mean age [±SD] 31 ± 5 years for case-patients vs. 30 ± 2 years for controls, t = −0.24, p = 0.81). Among case-patients with NiV infection and neighborhood controls, NiV case-patients were more likely than controls to have consumed raw date palm sap during the month before the case-patient’s illness (69% in case-patients vs. 30% in controls, matched odds ratio [mOR] 7.9, p = 0.01) and were more likely than controls to have been in the same room as case-patients (29% vs. 4%, mOR undefined, p<0.01) or to have touched (29% vs. 4%, mOR undefined, p value undefined) case-patients.

**Table 2:** Bivariate analysis of risk factors for Nipah virus infection, Faridpur, Bangladesh 2010*

Risk factor	No. (%) case-patients with risk factors, n = 15	No. (%) controls with risk factors, n = 58	mOR (95% CI)	p value
Male sex	8 (53)	26 (46)	1.3 (0.4–4)	0.62
Climbed tree	5 (33)	16 (29)	1.6 (0.7–3.7)	0.24
Physical contact with living animal				
Cow	11 (73)	32 (57)	2.5 (0.6–9.9)	0.2
Goat	5 (33)	26 (46)	0.4 (0.1–2.2)	0.3
Pig	0	1 (2)	Undefined	Undefined
Chicken	10 (67)	39 (70)	0.9 (0.3–2.9)	0.83
Duck	8 (53)	20 (36)	1.9 (0.7–5.8)	0.23
Dog	1 (7)	4 (7)	0.9 (0.1–7.9)	0.91
Cat	1 (6)	7 (13)	0.4 (0.1–3.9)	0.45
Fruit bat	0	0	Undefined	Undefined
Physical contact with sick animal				
Cow	0	4 (7)	Undefined	Undefined
Goat	1 (7)	1 (2)	0.9 (0.4–2)	0.76
Chicken	0	9 (16)	Undefined	Undefined
Duck	1 (7)	3 (5)	1.2 (0.1–15)	0.89
Ate any animal that had been sick	1 (7)	3 (5)	1.2 (0.1–15)	0.89
Had seen bats in or around residence at night	12 (80)	39 (70)	1.2 (0.1–15)	0.89
Drank raw DPS	9 (69)	17 (30)	7.9 (1.6–40)	0.012
Drank DPS before 9:00 am	8 (53)	16 (100)	Undefined	Undefined
DPS was				
Purchased	2 (20)	5 (33)	0.4 (0.04–4.8)	0.49
Given	2 (20)	4 (27)	Undefined	Undefined
Collected by a family member	6 (67)	5 (33)	Undefined	Undefined
Consumption of raw DPS during month before onset of illness, per day				
0	5 (33)	40 (71)	1	
<1 glass	4 (27)	4 (7)	32 (2.1–474)	0.01
1 glass	3 (20)	9 (16)	4.3 (0.6–29)	0.13
>1 glass	3 (20)	3 (5)	22 (1.2–404)	0.04
Is DPS harvester by profession	4 (27)	4 (7)	8.7 (0.9–83)	0.06
Household member harvests DPS by profession	4 (27)	4 (7)	8.7 (0.9–83)	0.06
Household distributes or sells DPS	4 (27)	5 (9)	10 (1.1–100)	0.04
Ate fruit				
Banana	11 (73)	33 (60)	1.9 (0.5–6.5)	0.32
Boroy/plum	7 (47)	29 (53)	0.8 (0.2–3.1)	0.75
Papaya	8 (53)	29 (52)	1.1 (0.3–3.2)	0.95
Sofeda	3 (20)	14 (25)	0.7 (0.1–3.7)	0.67
Kamranga	0	6 (11)	Undefined	Undefined
Guava	4 (29)	14 (25)	1.4 (0.2–8.4)	0.71
Tamarind	1 (7)	8 (14)	0.4 (0.4–3.7)	0.42
Custard apple	1 (7)	1 (2)	Undefined	Undefined
Visited another subdistrict	5 (33)	14 (25)	1.6 (0.4–5.7)	0.47
Touched someone with fever and altered mental status who died later	4 (29)	2 (4)	Undefined	Undefined
Was in same room as someone with fever and altered mental status who died later	4 (29)	2 (4)	Undefined	Undefined

*mOR, matched odds ratio; DPS, date palm sap.

## Discussion

During 2010, we identified an outbreak and several sporadic cases of encephalitis caused by NiV infection. Two case-patients from the outbreak and 3 patients with sporadic cases had IgM against NiV in serum and NiV RNA in oropharyngeal swab samples by conventional and real-time reverse transcription PCR (14). We could not collect biological specimens from 3 probable outbreak case-patients, including the source case-patient; however, the onsets of illness of patients with confirmed and probable cases were within 3 weeks of each other in an area where NiV outbreaks have been repeatedly confirmed over the past decade (2,3,7–10,15,16). Clinical features of fever, evidence of brain involvement, and rapid progression to death were also consistent with previous NiV outbreaks (2,3,8–10,12).

The first 4 case-patients of the initial phase of the outbreak, and 6 of 8 of the patients with sporadic cases, apparently contracted NiV infection by drinking raw date palm sap contaminated with NiV by *Pteropus* bats, an exposure that has been linked to NiV infection in previous outbreaks (3,17).

The remaining 4 case-patients from the outbreak probably acquired NiV infection from physical contact with the source case-patient. Such person-to-person transmission has been observed in prior NiV outbreaks in Bangladesh (7–10). The 2 generations of transmission are reflected in the 2 peaks in the epidemiologic curve (Figure 2). Among the second-generation cases, a novel finding was the transmission of NiV from the corpse of the source case-patient to 2 persons who had contact with the corpse before burial. This is the most plausible transmission pathway, because they did not have known exposures to living persons with encephalitis and had no history of drinking raw date palm sap. Because NiV is found in the respiratory secretions of NiV case-patients (14), case-patient G may have had intimate hand and facial contact with the corpse’s respiratory secretions while performing ritual purification. Consistent with the culturally prescribed method of ritual bathing of a corpse, case-patient H did not wear a mask or gloves during cleansing of the corpse’s orifices. He only used 3 pieces of cloth and his bare hands, which then were almost certainly contaminated with NiV. Case-patient H also likely touched his face or nose during or after the ritual purification. Persons commonly touch their own faces subconsciously, and 1 videotaped observational study found that persons touched their own eyes, nostrils, and lips 16 times per hour during normal activities (18). During Muslim ritual bathing, water is poured on the body (19). Thus, the water may have become contaminated with NiV and came in contact with case-patient H’s clothes and body. Similar to other infectious diseases, including severe acute respiratory syndrome and measles, the transmission efficiency of individual NiV case-patients varies (17,20,21). Case-patient A was an unusually efficient spreader of NiV, perhaps because of an unusually high concentration of NiV in his oral secretions.

The dead bodies of all NiV-infected patients who are Muslim in Bangladesh have undergone the same process of ritual bathing, but to our knowledge, corpse-to-human transmission has not been previously. In other NiV outbreaks when NiV infection developed in family members, many persons had contact with the source case-patient during illness and when preparing the corpse, so we were unable to separately assess corpse-to-person transmission. This investigation suggests that occasional NiV transmission could occur during the Muslim ritual purification of a corpse before burial.

This study also documents the death of a physician in Bangladesh from NiV encephalitis after he cared for NiV-infected patients with encephalitis in the surveillance hospital. The physician’s colleagues and roommates did not report any history of his drinking raw date palm sap during the month preceding onset of illness. Although the physician had contact with oral secretions of several meningoencephalitis patients during the outbreak, the genetic sequence of NiV found in the physician was distinct from those of 2 hospitalized NiV-infected case-patients who were positive for NiV by reverse transcription PCR (14). Indeed, none of the 3 hospitalized patients with confirmed NiV infection was likely to have been the source of the physician’s infection. The duration between onset of illness of the physician and his contact with confirmed NiV case-patients was beyond the range of the 6- to 11-day incubation period for NiV (12,17). During the assumed time of exposure to NiV, he cared for patients in the adult medicine ward; some of them may have had NiV infections that were missed by hospital surveillance. However, we did not identify any patient who met the case definition for meningoencephalitis in that ward 6–11 days before onset of the physician’s illness. The clinical spectrum of human NiV infection in Bangladesh also includes patients who sought treatment with respiratory disease as the primary manifestation (12), and surveillance may have missed any NiV-infected persons on the ward with this clinical manifestation. Another line of evidence suggests that an unidentified NiV-infected patient was hospitalized on that adult medicine ward at that time. One patient with a sporadic case, who visited FMCH as a family caregiver, also provided care for several patients in the men’s medicine ward during the same days that the physician attended to patients on that ward. This case-patient may have come in close physical contact with the same unidentified NiV-infected case-patient as the physician.

During 2001, health care workers were infected by NiV in Siliguri, India. Among 66 infected persons, 45 case-patients were hospital staff or family caregivers attending to the patients, and 11 patients were infected from an unidentified, hospitalized index case-patient (22). However, during an NiV outbreak in Bangladesh in 2004, health care providers (using minimal personal protective equipment [PPE] and with substantial exposure to NiV case-patients) had no evidence of having acquired NiV infection (11). During 2010 in Faridpur, NiV was transmitted from person to person in community and hospital settings. The observed differences in risk for person-to-person transmission between outbreaks suggest that NiV strains may differ in their proclivity for person-to-person transmission.

Because NiV infection is not the major cause of acute meningoencephalitis in Bangladesh, and because most persons who contract NiV infection have died by the time a diagnosis is made, it is difficult to identify a strategy to prevent person-to-person transmission that could be consistently applied to NiV-infected case-patients. Strategies to reduce care providers’ exposure to respiratory secretions could prevent a broad array of saliva-transmitted infections, including NiV encephalitis. Prevention approaches to reduce corpse-to-person transmission of NiV and other potentially fatal respiratory secretion-transmitted viruses should focus on minimizing exposure to saliva and other bodily fluids from the body of a person who died of severe febrile illness. Wearing gloves and a mask during the handling and washing of a dead body before burial would not be feasible in low-income communities, where the annual total per capita spending on health is US $12 per person per year (23). Research to identify culturally acceptable cost-effective approaches that can be consistently implemented in low-income settings, for example, washing hands thoroughly with soap and water immediately after corpse contact, could save lives.

This report of nosocomial transmission of NiV to a health care worker in Bangladesh after caring for NiV-infected patients highlights the risk of working without PPE. Barriers to developing an appropriate prevention strategy for nosocomial transmission of NiV in hospitals in Bangladesh include the following: inadequate supplies of PPE for hospital staff, absence of isolation wards, absence of handwashing facilities in hospital wards and physicians’ rooms, and inadequate training and monitoring for infection control (24). Because saliva is the most likely vehicle for transmission of NiV among care providers, implementation of standard and contact precautions (25) that have been culturally and economically customized to fit this setting could reduce NiV transmission. As a first step, we recommend that handwashing stations be established and consistently supplied with soap and water in every ward of the hospital for health care workers and patient attendants. Second, because laboratory diagnosis for NiV infection is not available during the initial evaluation of patients with meningoencephalitis syndrome, during NiV season all hospitals in NiV infection–prone areas should admit patients with meningoencephalitis syndrome into an isolation room or ward and routinely provide gloves and masks for health care workers when they are caring for meningoencephalitis patients. Patient attendants could reduce their exposure to patient saliva and respiratory secretions by frequent handwashing and by avoiding sharing food and beds with patients.
